# Group 2 Innate Lymphoid Cell Proportions Are Diminished in Young Helminth Infected Children and Restored by Curative Anti-helminthic Treatment

**DOI:** 10.1371/journal.pntd.0003627

**Published:** 2015-03-23

**Authors:** Norman Nausch, Laura J. Appleby, Alexandra M. Sparks, Nicholas Midzi, Takafira Mduluza, Francisca Mutapi

**Affiliations:** 1 Institute of Immunology and Infection Research, Centre for Immunity, Infection and Evolution, School of Biological Sciences, University of Edinburgh, Ashworth Laboratories, Edinburgh, United Kingdom; 2 National Institute of Health Research, Causeway, Harare, Zimbabwe; 3 University of Zimbabwe, Department of Biochemistry, University of Zimbabwe, Mount Pleasant, Harare, Zimbabwe; George Washington University Medical Center, UNITED STATES

## Abstract

**Background:**

Group 2 Innate lymphoid cells (ILC2s) are innate cells that produce the TH2 cytokines IL-5 and IL-13. The importance of these cells has recently been demonstrated in experimental models of parasitic diseases but there is a paucity of data on ILC2s in the context of human parasitic infections and in particular of the blood dwelling parasite *Schistosoma haematobium*.

**Methodology/Principal Findings:**

In this case-control study human peripheral blood ILC2s were analysed in relation to infection with the helminth parasite *Schistosoma haematobium*. Peripheral blood mononuclear cells of 36 *S*. *haematobium* infected and 36 age and sex matched uninfected children were analysed for frequencies of ILC2s identified as Lin-CD45+CD127+CD294+CD161+. ILC2s were significantly lower particularly in infected children aged 6–9 years compared to healthy participants. Curative anti-helminthic treatment resulted in an increase in levels of the activating factor TSLP and restoration of ILC2 levels.

**Conclusion:**

This study demonstrates that ILC2s are diminished in young helminth infected children and restored by removal of the parasites by treatment, indicating a previously undescribed association between a human parasitic infection and ILC2s and suggesting a role of ILC2s before the establishment of protective acquired immunity in human schistosomiasis.

## Author Summary

Understanding how immune responses are generated is critical for vaccine development. There are comparatively few studies on the interface between the innate and adaptive immune system in generating protective immune responses. Infections with helminth parasites, a cause of neglected tropical diseases, have a huge collective impact on public health in affected developing countries. Helminths are associated with a complex type 2 immune response mediated by cytokines characteristically produced by adaptive T helper 2 cells (T_H_2). However in recent years a newly described type of innate immune cells has been shown to produce T_H_2 cytokines. This cell type was subsequently called ‘Group 2 innate lymphoid cells’ (ILC2s). The importance of these cells has been demonstrated in experimental models of helminth infection as well as in allergic diseases. The present study describes changes in human ILC2s during the human helminth infection schistosomiasisas (bilharzia). Our study shows that the proportions of ILC2s, were lower in young, but not older infected children when compared to uninfected participants, suggesting a role for ILC2s before the establishment of helminth protective acquired immunity. Furthermore ILC2s were restored after curative treatment of the helminth infection. Human mechanistic studies will determine if the association between ILC2s and schistosome infection is causal or a marker of resistance to infection.

## Introduction

Innate lymphoid cells are a recently described cell type that has transformed our understanding of the role of innate immune responses in the generation of adaptive immune responses. Group 2 Innate lymphoid cells (ILC2s) produce the classical T_H_2 cytokines IL-5 and IL-13 [1–3] and have been shown to be crucial for protective immune responses in experimental helminth infection [4–6]. ILC2s play an important role as an early source of IL-13 and are crucial for timely worm expulsion in mice infected with the murine helminth parasite *Nippostrongylus brasiliensis*. However, to date there is only one study in humans which characterised ILCs in a small population of patients with diverse filarial infections [7].

The interaction of helminth parasites and the host immune system has been extensively studied in experimental models and naturally infected humans. In experimental models, the immune responses to helminths, including schistosome infections, is polarized towards a type 2 immune response (reviewed in [8–10]). However, research over the last decade has shown that the establishment of helminth infections requires modulation of host type 2 responses, which is mediated by several different mechanisms including regulatory T cells, regulatory B cells and the immuno-modulatory cytokines IL-10 and TGF-β [11–15]. To a large extent these observations have been supported by evidence from human studies [16–18]. Type 2 immune responses have been shown to involve innate immune cells including dendritic cells, basophils and alternatively activated macrophages [19–21]. However in human, T_H_2 responses to helminth infections are less pronounced [22, 23]. In addition, humans infected with helminths show alterations of cellular responses such as regulatory T cells, dendritic cells and CD4+ T Cell responses [16, 24, 25] and these responses vary with age (and thereby with history of infection with helminths). Thus, the specific characteristics of the human type 2 response to helminths are more complex than in experimental models meaning that observations from the experimental setting must be validated in natural human infections.

Human ILC2s are negative for lineage markers (Lin-), but positive for the hematopoietic cell marker CD45 [26]. IL-7 is essential for the development of ILC2s [5, 27] and therefore CD127 (IL-7Rα) is a main marker for characterising ILC2s. Furthermore human ILC2s express the ‘chemokine receptor homologous molecule expressed on T_H_2 cells’ (CRTH2 = CD294) a marker of T_H_2 cells [28] and expressed the NK cell receptor CD161 (NKR-P1A) [26], whereas CD117 (c-kit) is mainly expressed on ILC3 and only on a subset of ILC2s [2].

ILC2s were initially described in fetal and adult gut and lung, but have also been identified in peripheral blood [26], but the function of blood ILC2s has not yet been investigated in detail. Changes in proportions of blood ILC2s could reflect either a global modulation of ILC2s or changes in the potential to migrate to tissues.

ILC2s are dependent on the T_H_2-associated transcription factors GATA-3 [29, 30] and RORα [27, 31] and the ‘thymic stromal lymphopoietin’ TSLP enhances GATA-3 expression and subsequent production of IL-4, IL-5 and IL-13, especially if supplied in combination with IL-33, but less efficiently with IL-25 [32]. Changes of systemic IL-33 during helminth infection have been recently reported [33].

The presence of ILC2s in human peripheral blood has been demonstrated in healthy individuals and in patients with mild or severe asthma [34, 35]. Furthermore, in mouse models ILC2s as well as IL-25 and IL-33, have been shown to be involved in allergic immune responses [36–41].

The aim of this study was to determine if levels of blood ILC2s change in the context of a parasitic infection in a human population, thereby providing evidence that ILC2s are important in human parasitic diseases. The study focused on the helminth parasite of the genus *Schistosoma* (blood-flukes) which causes schistosomiasis, a neglected tropical disease affecting about 240 million people mainly in Sub-Saharan Africa [42]. The most prevalent form is urogenital schistosomiasis caused by *Schistosoma haematobium*. The heaviest burden of this helminthic disease occurs in children, who typically acquire infection in the first year of life [43, 44]. Infection accumulates with age, and in most populations peaks around the age of 9–14 years, followed by a decrease in infection [45, 46] which has been attributed to the development of protective acquired immunity [47, 48]. Therefore immune epidemiological analyses covering these different stages of infection have been shown to be important in the analysis of the immune response towards helminth infections [16, 24, 25].

In this study we describe for the first time, alterations of ILC2 proportions in human blood during natural infection with schistosomes in a heterogeneous population (different ages to capture the different infection dynamics of rising, peaking and declining infection levels) and the effect of removing the helminth parasites through curative anti-helminthic drug treatment on ILC2 proportions. To elucidate pathways involved in changes of ILC2s, levels of the ILC2 activating factors TSLP and IL-33 were analysed.

## Methods

### Ethical statement

The study received institutional and ethical approval from the Ethical Review Board of the University of Zimbabwe and the Medical Research Council of Zimbabwe respectively. Permission to conduct the study in the selected area was obtained from the Provincial Medical Director, the District Educational Officer and School Heads. The aims and procedures of the study were explained to participants and their parents/guardians (in the case of children) in the local language, Shona, with written informed consent and assent obtained from the participants or parents/guardian before enrolment into the study and before administration of the anti-helminthic drug. Participants were free to drop out at any time of the study and parents/guardian could withdraw their children from the study. After sample collection, anti-helminthic treatment with the standard dose of praziquantel was offered to all participants and administered by a local physician.

### Study area and design

This study was conducted in Magaya village in the Murehwa district of the Mashonaland East Province of Zimbabwe (31°91’E; 17°63’S) as part of a larger study investigating the immuno-epidemiology of human urogenital schistosomiasis of which several aspects have been published [24, 25, 49, 50]. Samples used within this study were collected between September and November 2008. Previous studies in this area and national surveys indicated low prevalence’s of *S*. *mansoni* and soil-transmitted helminths (STH), whereas the *S*. *haematobium* prevalence was high (>50%) [51, 52]. The area is mesoendemic for *Plasmodium* infection [53].

Magaya is a rural village where subsistence farming is the predominant occupation. Due to lack of safe water and sanitation facilities, participants are in frequent contact with fresh water, which harbours the parasite life stage (cercariae) infective to the human host. Frequency of water contacts of participants, their history of anti-helminthic treatment and residential history in the schistosome endemic area were documented by questionnaires.

### Study group

To be included in the cross-sectional part of the study, participants had to meet the following criteria: a) been lifelong residents of the study area, b) not have previously received anti-helminthic treatment, c) be negative for *S*. *mansoni*, STH, *Plasmodium falciparum* (ensuring that the confounding effects of these parasites were excluded from the study) and HIV, d) have provided at least two urine and two stool samples on consecutive days for parasitological analysis and a blood sample sufficient for PBMC and plasma isolation. From the participants who fulfilled these criteria a group of 72 people were selected covering an age range of 6–18 years with 24 people in each of following three age groups: 6–9, 10–13, 14–18 years. Within each age group equal numbers of uninfected and infected people were selected providing a *S*. *haematobium* prevalence of 50%. Furthermore participants were age and sex matched between the uninfected and infected groups. Egg positive samples were chosen to have comparable infection intensities between the three age groups. The resulting study group is shown in Table 1.

**Table 1:** Description of the selected study group.

Age group (years)	*S*. *haematobium* Status	Sample size (N)	Mean age (years)	*S*. *haematobium* Infection intensity: Mean / Median (range)	M/F
6–9	egg ve-	12	7.83	0	6/6
	egg ve+	12	7.92	28.3 / 16.0 (2.7–158.3)	6/6
	Total	24	7.88	14.1 / 1.3 (0–158.3)	12/12
10–13	egg ve-	12	11.58	0	4/8
	egg ve+	12	12.75	34.4 / 23.3 (3.0–132.0)	7/5
	Total	24	12.17	17.2 / 1.5 (0–132.0)	11/13
14–18	egg ve-	12	15.33	0	5/7
	egg ve+	12	14.92	31.6 / 21.5 (1.7–100.3)	5/7
	Total	24	15.12	15.8 / 0.8 (0–100.3)	10/14
Total (6–18)	Prevalence 50%	72	11.64	15.2 / 0.8 (0–158.0)	33/39

All participants were negative for HIV, soil transmitted helminths and *S*. *mansoni*; M—male, F—female, infection intensity: eggs / 10 mL urine; ve- negative, ve+ positive

The effect of removing the parasites on ILC2s and levels of the activating factors TSLP and IL-33 were determined in a cohort study where participants were treated with the anti-helminthic drug praziquantel at the recommended dose of 40 mg/mL and followed up 6 weeks later (before re-infections reach patency) as recommended [54]. To be included in this study participants had to meet the following criteria a) been included in the cross-sectional study described above, b) been positive for infection with *S*. *haematobium* parasites at baseline, c) been treated with the anti-helminthic drug praziquantel, d) have provided at least two urine and two stool samples for a parasitology check and have been confirmed negative for infection with *S*. *haematobium* parasites 6 weeks after treatment, e) provided a blood sample for isolation of PBMC and plasma at the 6 week post-treatment survey. Twelve participants, 9 males and 3 females, with a mean age of 9.58 years (range 6–13 years) and a mean infection intensity of 43.0 eggs/10 mL (range 3.0–158.3) before treatment fulfilled these criteria.

### Parasitology, blood collection and isolation of PBMC

At least two urine and two stool samples were collected over three consecutive days (between 9am and 1pm). Infection with *S*. *haematobium* was determined by filtration of 10 mL urine and microscopic analysis following the standard urine filtration procedure [55]. Infection with *S*. *mansoni* and STH was determined in stool samples using the Kato-Katz method [56], with the results confirmed in a random subset of stool samples by the formol-ether concentration technique [57].

Up to 20 mL of venous blood was collected into heparinised blood collection tubes and a further five mL into EDTA coated tubes. Blood was analysed for HIV using the rapid test ‘DoubleCheckGold^TM^ HIV 1&2’ (Orgenics) and positive samples were re-tested using ‘Determine HIV ½ Ag/Ab Combo’ (InvernessMedical). Blood smears were stained with Giemsa, microscopically examined for *Plasmodium falciparum* and checked using a serological test (Paracheck-PF®, Orchid Biomedical Systems). Heparinised blood was used for the isolation of PBMCs through density gradient centrifugation Lymphoprep^TM^ (Axis-Shield). PBMC were cryopreserved in 10% DMSO (Sigma) and 90% fetal calf serum (Lonza).

### Phenotyping ILC2 and plasma analysis

1x10^6^ PBMCs were surface stained using the following anti-human antibodies: FITC-conjugated lineage cocktail (BD Bioscience) containing CD3, CD14, CD16, CD19, CD20, CD56, FITC-conjugated anti-CD11c (clone 3.9), FITC-conjugated anti-CD123 (clone 6H6), e450-conjugated anti-CD161 (HP-3G10), APC-e780-conjugated anti-CD127 (eBioRDR5; all eBioscience), VioGreen-conjugated anti-CD45 (clone 5B1), APC-conjugated anti-CD117 (A3C6E2), PE-conjugated anti-CD294 (BM16; all Miltenyi Biotec). At least 4x10^5^ stained PBMCs were acquired on a FACSCantoII^TM^ (BD Bioscience) and analysed using FlowJo7 software (TreeStar). Previous experiments confirmed that cryopreserved PBMCs did not include any basophils and mast cells using this phenotyping approach. Plasma levels of TSLP and IL-33 by ELISA were analysed using commercially available kits (eBioscience and RnD systems respectively). IL-4, IL-5 and IL-13 were measured using established protocols [50, 58].

### Statistical analysis

Since the data did not meet the assumptions of parametric tests, all statistical analyses were carried out using non-parametric tests. For the comparison of two groups (egg negative *versus* egg positive, male *versus* female), Mann-Whitney *U* test was applied, for multiple groups the Kruskal-Wallis test followed by a post-hoc comparison (Mann-Whitney *U* test with Bonferroni correction) was used. For comparison of paired data (pre- *versus* post-treatment) the Wilcoxon signed-rank test was applied. Correlations were tested using a Spearman's rho analysis. All statistical analysis was carried out using IBM SPSS v19 and p-values were taken as significant if ≤ 0.05.

## Results

### Characterisation of human ILC2s

**Fig 1:**
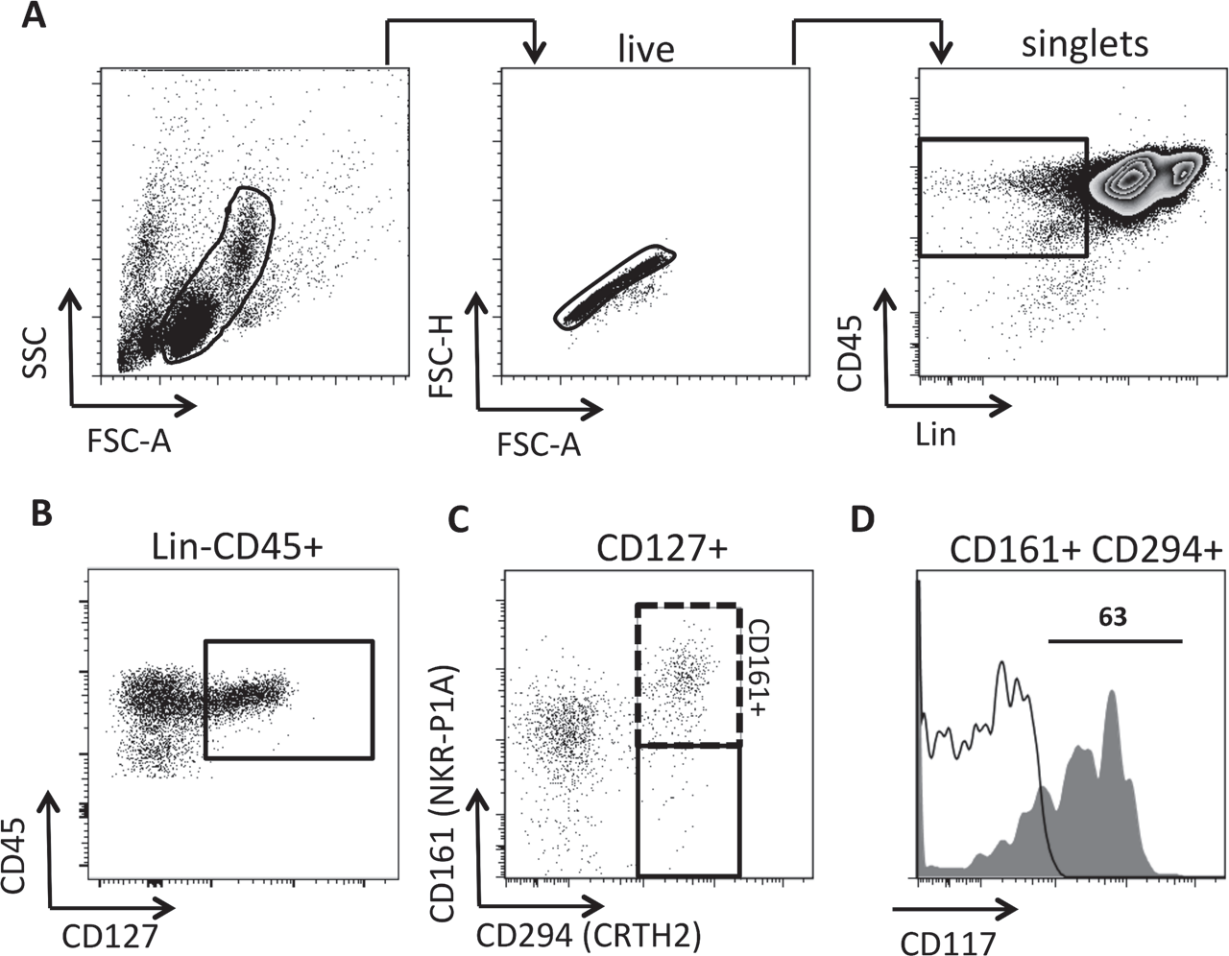
Characterisation of human ILC2 by flow cytometry. (A) Human PBMC were gated on live cells using forward (FSC) and side (SSC) scatter. Gated live cells were analysed using the area against height of the FSC to discriminate singlet’s from duplicates. Singlet’s were subsequently analysed by a lineage cocktail (Lin) and CD45 to gate on Lin-CD45+ cells (as indicated in the box) (B). Lin-CD45+ cells were gated on CD127+ cells, which were analysed for CD294 and CD161 (C). CD127+CD294+CD161+ were analysed for CD117 (grey histogram) versus isotype control (open histogram) (D). Flow charts as presented are from the PBMC analysis of a 9 year old female who was negative for *S*. *haematobium* eggs.

To identify human peripheral blood ILC2s, Mjösberg and co-workers [26] depleted PBMCs from T cells, B cells and monocytes, followed by a flow cytometric analysis of the remaining cells. This approach was not practical here, due to a much larger sample size used in the present study, and as cell numbers were limited. We therefore directly stained whole PBMCs with a lineage cocktail, which also included the NK cell marker CD56. PBMCs were gated on live cells and duplicates excluded (Fig. 1A). Gated single cells were analysed by a Lin cocktail and CD45, which showed a Lin- negative population expressing CD45. Within this population a CD45^hi^ and a CD45^int^ population, could be clearly distinguished.

To identify human ILC2s, Lin-CD45+ PBMCs were gated on CD127+ (Fig. 1B). Within Lin-CD45+CD127+ cells a defined population of CD294+CD161+ cells could be identified (Fig. 1C). CD294+CD161+ cells contained both a CD117+ and a CD117- subset (Fig. 1D). In reverse CD127+CD117+ ILCs did not exclusively define CD294+ and CD161+ cells (Median of CD294+CD161+ in CD127+CD117+: 45.6, range 13.1–74.7) and it is known that CD117+ compromise both ILC2s and ILC3s. Of note, CD127+CD294+CD161+ subsets are all CD45^hi^. In summary, although not completely homogenous Lin-CD45+CD127+CD294+CD161+ cells define a population of human blood ILC2s, hence CD127+CD294+CD161+ ILC2s were used in subsequent analyses.

### Proportions of ILC2s are diminished during parasitic helminth infection

**Fig 2:**
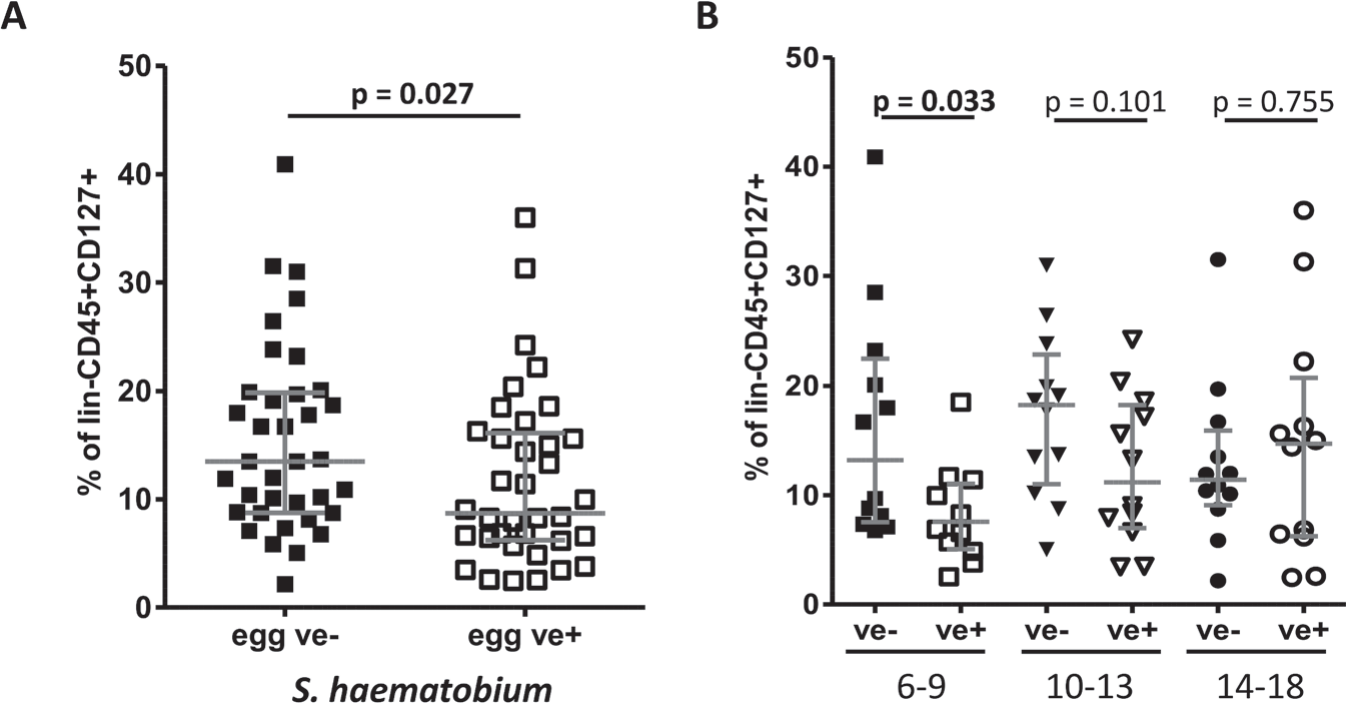
Proportions of ILC2s identified by CD127+CD294+CD161+ are lower in *S*. *haematobium* egg positive young children. (A) The participants were compared between *S*. *haematobium* egg negative (ve-, N = 36) and egg positive (ve+, N = 36) individuals by a non-parametric Mann-Whitney *U* test. (B) The cohort was divided into three age groups (age in years) and egg positive (ve+, open symbols) were compared to egg negative (ve-, closed symbols) people. Grey lines indicate median and the interquartile range. ILC2s proportions were analysed using a Kruskal-Wallis test followed by a multiple comparison of egg positive *versus* egg negative children.

Next, the effect of current schistosome status on the proportions of blood ILC2s was analysed. The analyses showed that CD127+CD294+CD161+ ILC2s were all significantly lower in schistosome-infected individuals (p = 0.027) when levels of these cells were expressed as percentages of lin-CD45+CD127+ (Fig. 2A). This was also true if ILC2s were expressed as percentages of lin-CD45+ (Median: 4.8 egg negative (ve-), 3.1 egg positive (ve+) p = 0.044). In addition proportions of ILC2s were negatively correlated to the intensity of infection determined by egg count (r = −0.300, p = 0.005 using non-parametric Spearman correlation). Of note, CD127+CD117+ ILCs show a comparable pattern (Median: 13.7 egg ve-, 7.5 egg ve+, p = 0.001).

To determine if these differences were consistent across age groups undergoing different schistosome infection dynamics, the population was divided into three age groups, 6–9, 10–13 and 14–18 year olds reflecting rising, peaking and declining infection levels. This analysis showed that ILC2s in infected participants were significantly lower than those in uninfected people of the youngest age group (Fig. 2B). This difference was not significant in children aged 10–13 years and proportions of ILC2s were comparable in the oldest age group aged 14–18 years. Comparable results were obtained if ILC2s levels were correlated to infection intensity. The youngest age group showed a strong negative correlation between infection intensity and ILC2 proportions (r = −0.483, p = 0.008), whereas a weaker negative correlation was observed in 10–13 old participants (r = −0.403, p = 0.025) and with no significant correlation in the 14–18 years old (r = −0.045, p = 0.418).

CD127 (IL-7Rα) was used for identification of ILC2s, allowing the actual expression levels (MFI) of CD127 on ILC2s to be measured. Expression levels of CD127 did not show significant variation between infected and uninfected people for all age groups (6–9 years: p = 0.514; 10–13 years: p = 0.114; 14–18 years: p = 0.551).

Of note, levels of the systemic effector T_H_2 cytokines IL-4, IL-5 and IL-13 did not show any association with peripheral ILC2s (i.e. ILC2s to IL-4: r = −0.113 (p = 0.346); to IL-5 r = 0.138 (p = 0.248 and to IL-13 r = 0.016 (p = 0.894) using non-parametric Spearman correlation). Systemic T_H_2 cytokines were positively associated to infection intensity only in the oldest age group (IL-4: r = 0.488, p = 0.008; IL-5: r = 0.400, p = 0.027; IL-13: r = 0.497, p = 0.007). Detailed data for the T_H_2 cytokines are presented in S1 Fig with IL-5 and IL-13 only significant in children 14–18 years of age.

### Curative anti-helminthic treatment restores ILC2 levels

**Fig 3:**
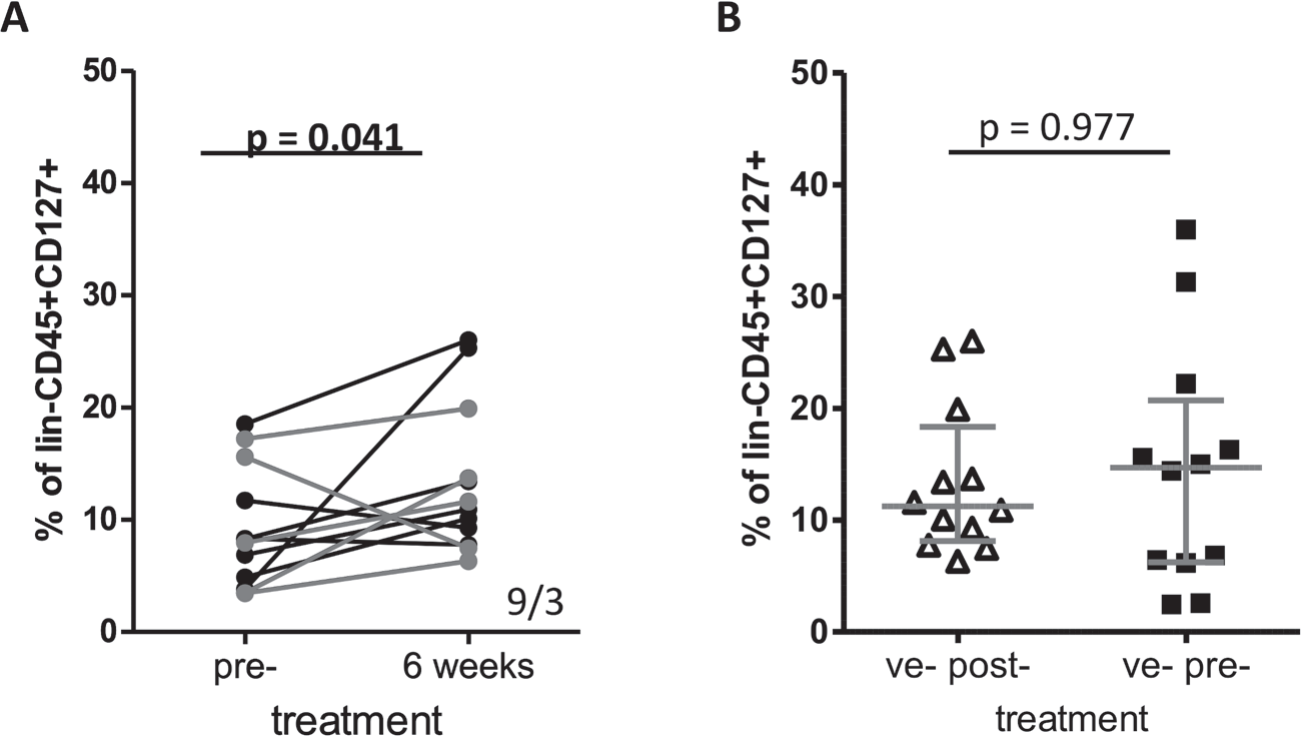
Proportions of ILC2s increase 6 weeks after curative anti-helminthic treatment with praziquantel in children aged 6–13 years. (A) The proportions of ILC2s of 12 individuals who were positive pre-treatment and had cleared *S*. *haematobium* infections 6 weeks post-treatment were analysed using a non-parametric Wilcoxon signed-rank test. Numbers in the lower-right corner indicate positive/negative ranks. Grey lines are individuals 10–13 years whereas black lines present children 6–9 years of age. (B) The proportions of ILC2s compared between the 12 children aged 6–13 years who had completely cleared their *S*. *haematobium* infections 6 weeks post-treatment (open triangles) and the 12 participants aged 14–18 years who were already *S*. *haematobium* negative before treatment (black squares). Grey lines indicate median and the interquartile range.

To test if the differences in the ILC2 proportions were related to the presence of schistosome parasites, schistosome adult worms were removed by curative treatment and analysed in 12 children aged 6–13 years who were *S*. *haematobium* positive prior to treatment and had cleared infection 6 weeks later were followed up and proportions of ILC2s were determined. The overall proportions of their ILC2 cells increased significantly post-treatment as shown in Fig. 3A.

Older egg negative participants have putatively developed a protective immune response and have been referred to in other studies as endemic normal and a high worm specific IgE/IgG4 ratio is a marker for schistosome-specific resistance [25]. Curative treatment by chemotherapy has been shown to accelerate the development of a protective immune response [59], hence post-treatment levels of ILC2 were compared to egg ve- individuals aged 14–18 years. Post-treatment, levels of ILC2 rose up to levels observed in untreated 14–18 year old participants who were egg negative prior to treatment (Fig. 3B).

After dividing these 12 children into two groups of 6–9 and 10–13 years of age, ILC2s increased in 5 out of 7 and in 4 out of 5 children, respectively.

The increase of ILC2 levels were inversely related to the pre-treatment levels of these cells, with the lowest pre-treatment proportions exhibiting the highest magnitude of change post-treatment. We have previously reported this phenomenon in changes in antibody levels in children exposed to *S*. *mansoni* infection [59].

### Curative anti-helminthic treatment increases TSLP levels

**Fig 4:**
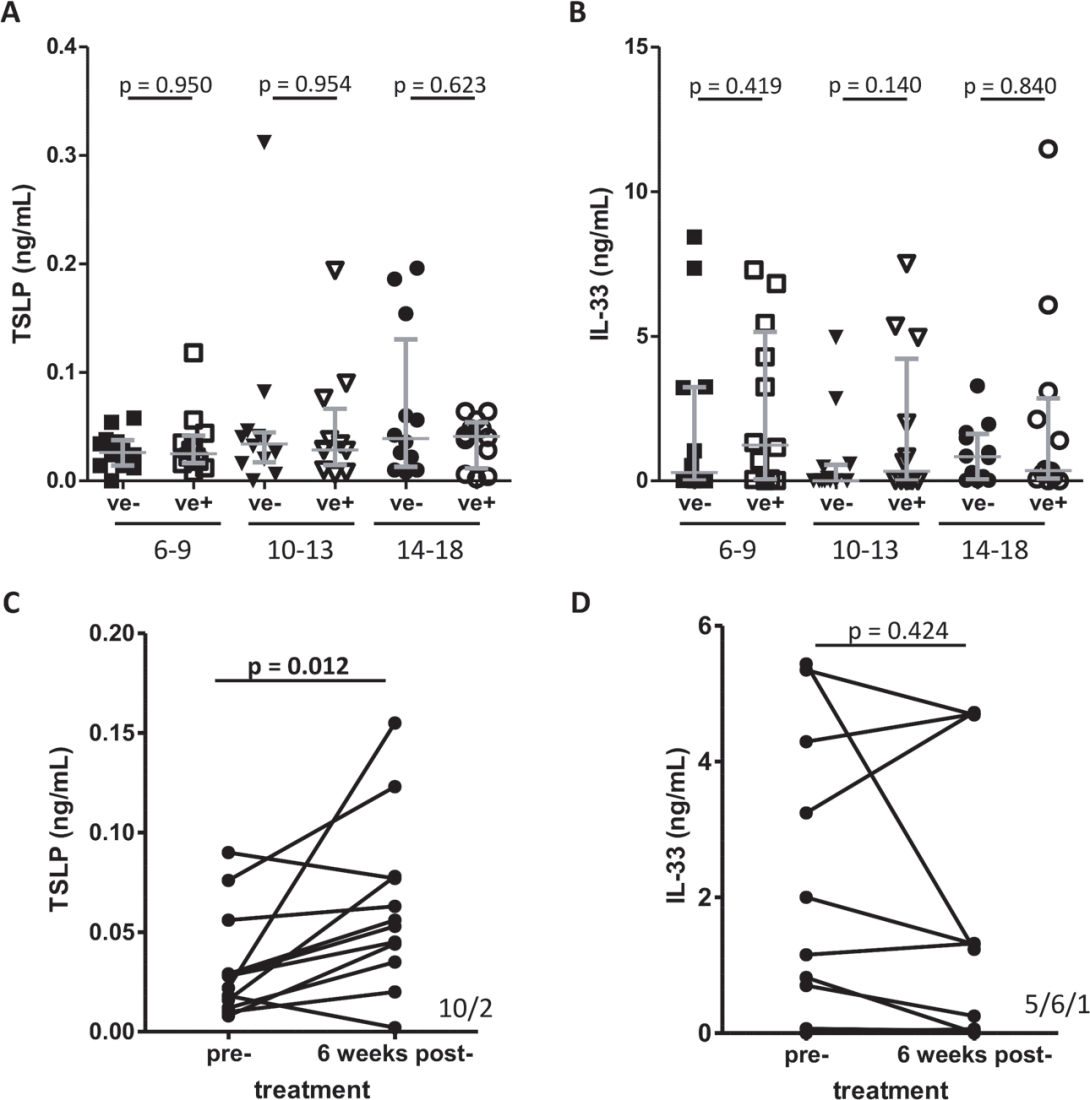
Comparison of plasma TSLP and IL-33 levels in relation to *S*. *haematobium* infection. Plasma levels of (A) TSLP and (B) IL-33 were measured by ELISA and data were divided into three age group and *S*. *haematobium* egg negative (ve-, closed symbols) compared to egg positive (ve+, open symbols) individuals. Grey lines indicate median and the interquartile range. Levels were compared using a Kruskal-Wallis test followed by a multiple comparison of egg positive *versus* egg negative children. Plasma levels of (C) TSLP and (D) IL-33 from 12 individuals (as described for Fig. 3) were compared pre- and 6 week’s post-treatment. P-values are obtained from a Wilcoxon signed-rank test and positive/negative/unchanged ranks are shown in the lower-right corner of panel (C) and (D).

Maintenance and activation of ILC2s has been reported to depend on the cytokines IL-25, IL-33 and TSLP. Pre-treatment plasma levels of IL-33 and TSLP were measured, but no significant difference was observed when egg negative and egg positive children were compared regardless of the age group investigated (Fig. 4A, B). However TSLP levels increased significantly 6 weeks after curative treatment (Fig. 4C). In contrast, the level of IL-33 remained unchanged following treatment (Fig. 4D).

## Discussion

In parasitic infections, ILC2s have been described in experimental models of helminth infections demonstrating their role as an early source of the cytokine IL-13 and in worm expulsion [4, 6, 60–62]. Given these results, this study aimed to determine if ILC2s populations are altered during human parasite infections using an immuno-epidemiological approach reflecting the complex dynamics during human parasite infections and specifically focusing on young children with accumulating infection. Hence, included in the study are young children who have yet to acquire schistosome infection (negative for schistosome eggs and parasite-specific antibodies), infected young and older children (schistosome egg positive) and older children putatively resistant to schistosome infection (schistosome egg negative but positive for schistosome-specific IgG and IgE [63]).

First, this study confirms that ILC2s can be identified in human peripheral blood using Lin-CD45+CD127+ cells in combination with CD161+ and CD294+ in a population of young African children.

This study, shows for the first time that proportions of human blood ILC2s are diminished during an infection with trematodes. The strongest effect was observed in the youngest age group. Reductions in proportions of ILC2s could theoretically arise in two ways; due to a reduction in generation/maintenance of the cells in people infected with *S*. *haematobium* parasites. Alternatively, ILC2s could migrate and accumulate at the site of infection or to the tissues where eggs get trapped, initiating a localised immune response thereby leading to a reduction of the cells in peripheral blood. Furthermore reduced levels of CD127 could indicate a reduced responsiveness to IL-7 and thereby ILC2 survival. However, expression levels did not show any variation which supports reduced responsiveness to IL-7. In this present study ILC2s are quantified as proportions. Changes in ILC2 proportions due to an increase of other cell types within Lin-CD45+ cells cannot be excluded. However, our results are consistent with those from a previous study on blood ILC2s, indicating that in a T_H_2 mediated disease, ILC2s are lower in patients compared to controls [35]. In contrast a recent study showed in 21 people with diverse filarial infections (*Loa loa*, *Wuchereria bancrofti* and with *Onchocerca volvulus*) an increase of ILCs characterised by CD127+CD117+ [7]. However infected people were aged 25–66 years and therefore significantly older than even in the oldest age group investigated in this study, in which ILC2s were also slightly higher in egg positive compared to egg negative people, which may become significant if older people analysed.

Interestingly, reduced ILC2s were mainly observed in the youngest children, whereas in older children (14–18 years) there were no differences between egg negative and positive individuals. These differences are likely to be related to the dynamics of infection and the immune response due to ILC2s may have different dynamics compared to the development of protective acquired immunity. Age-related changes of cellular, antibody and cytokine pattern in the context of schistosome infection have been reported previously [24, 64, 65] and such pattern are supported by theoretical models of helminth infections [66]. These changes have been taken to represent population changes from susceptibility to infection the development of protective acquired immunity [66]. Parasitological and immuno-epidemiological studies show that the egg negative children in the youngest age group in this population have yet to acquire infection (no parasite specific—IgM responses which are indicative of exposure to parasite life stages). The remaining study population are either currently infected (indicated by the presence of parasite eggs) or have had previous infection and are now putatively immune (indicated by the absence of parasite eggs in combination with the presence of parasite-specific immune responses associated with resistance to infection such as anti-worm antibodies (IgE, IgG1, IgE/IgG4 ratio) and parasite specific cellular responses (IL-5) as previously reported [25, 58, 67]). Thus, in this population, the youngest egg negative children are the only ones who have not yet experienced a schistosome infection, and the observed changes in ILC2s in this age group upon infection, suggests that these cells are important in the early phase of the immune response, when the T_H_2 pathway is initially triggered, rather than in the later phases of infection, when T_H_2 responses become established. This is consistent with our results from studies of the myeloid dendritic cells in this population [24], which show a similar pattern. Indeed it has been shown that ILC2s, in particular, provide an early source of IL-13 during experimental helminth infection [4]. Dynamic changes in the immune response to schistosomes are well documented and age-related changes of cellular immune responses have been previously reported in this and other study populations [16, 24, 25].

Clearance of *S*. *haematobium* infection following curative treatment with the anti-helminthic drug praziquantel caused an increase in ILC2 proportions, recovering to levels comparable to egg negative 14–18 year old participants, who have already developed natural protective immunity against parasites [25]. This is in keeping with earlier studies showing that chemotherapy accelerates the development of protective acquired immunity against schistosomes [59] as well as studies from this population showing that chemotherapy induced cellular responses associated with resistance against re-infection [50]. However due to the small sample size post-treatment, this result requires further verification.

Both IL-33 and TSLP are important in ILC2 activation and propagation. There was no significant association with systemic levels of IL-33 or TSLP with schistosome infection prior to treatment. Hence systemic levels of these cytokines do not explain the alterations in blood ILC2s and do not indicate a different activation status. However, if changes in ILC2 proportion are due to migration, IL-33 and TSLP might still play a role if regulated locally (in which case local levels of the cytokines would not necessarily be reflected systemically). We have obtained supportive evidence for the importance IL-33 by analysing the effect of a specific IL-33 single nucleotide polymorphisms (SNP) showing that allele variation influences schistosome infection intensity without influencing systemic IL-33 levels (manuscript in preparation).

In addition, systemic T_H_2 cytokines IL-4, IL-5 and IL-13 did not show significant associations with peripheral ILC2s. However, if changes in ILC2s are due to migration, ILC2 activation could be still initiated locally and play an important role in the immune response. This is supported by the finding that systemic T_H_2 cytokines are only associated to infection in the oldest age group, indicating that a classical T_H_2 response is more important and reflected on systemic levels in older chronically infected individuals. Further studies are required to analyse the activation status of ILC2s in infected people relative to their expression levels of GATA-3, IL-5 and IL-13 as well as receptors for IL-33 and TSLP. Recently is has been reported that in mouse models IL-9 and amphiregulin are important in the function of ILC2s [68, 69]. However, data in humans are sparce and both IL-9 and amphiregulin were not incorporated when this study was carried out.

Increased levels of TSLP were observed 6 weeks after treatment, which suggests that TSLP may be involved in the increase of ILC2s following treatment, but no change in IL-33 was observed after treatment. This latter result contradicts previous reports of an increase in IL-33 several weeks after treatment [33]. Further studies are required to clarify the role of IL-33 in human infections with *S*. *haematobium* and should also include IL-25, which was not analysed during this study due to limitations in blood levels that could be collected safely from the younger children.

In summary, this is the first study to report peripheral blood ILC2s being diminished in young children suggesting that ILC2s may play a role in the immune responses to human helminth infection before the establishment of schistosome protective immunity. Clearly further mechanistic studies are required to determine how ILC2 levels are diminished in helminth infection and how they are restored following removal of the helminth infection by curative treatment. Nonetheless, this study validates the paradigms demonstrated in experimental studies of an association between parasitic infection and ILC2 cells.

## Supporting Information

S1 Checklist STROBE checklist—case-control study. (PDF)

S1 Fig Comparison of levels of systemic T_H_2 cytokines. IL-4 (A), IL-5 (B) and IL-13 (C) were analysed by ELISA and data devided by age group and *S*. *haematobium* egg negative (ve-, closed symbols) are compared to egg positive (ve+, open symbols). Grey lines indicate Median and the interquartile range and levels were compared using a Kruskal-Wallis test followed by a multiple comparsion.
(TIF)
