# Supplementary material for: Group 2 Innate Lymphoid Cell Proportions Are Diminished in Young Helminth Infected Children and Restored by Curative Anti-helminthic Treatment
Source: PLoS Negl Trop Dis. 2015 Mar 23;9(3):e0003627. doi: 10.1371/journal.pntd.0003627 (PMC4370749; doi:10.1371/journal.pntd.0003627)
Supplement: S1 Checklist — (PDF) [file pntd.0003627.s001.pdf]

STROBE Statement—Checklist of items that should be included in reports of ***case-control studies***

	Item No	Recommendation
**Title and abstract**	1	*(a)* Indicate the study's design with a commonly used term in the title or the abstract Abstract/Methodology: "**In this case-control study** human peripheral blood ILC2s were analysed in relation to infection with the helminth parasite *Schistosoma haematobium*.
		(*b*) Provide in the abstract an informative and balanced summary of what was done and what was found **see abstract:** Peripheral blood mononuclear cells of 36 *S. haematobium* infected and 36 age and sex matched uninfected children were analysed for frequencies of ILC2s identified as Lin-CD45+CD127+CD294+CD161+. ILC2s were significantly lower particularly in infected children aged 6-9 years compared to healthy participants. Curative anti-helminthic treatment resulted in an increase in levels of the activating factor TSLP and restoration of ILC2 levels.
**Introduction**		
Background/rationale	2	Explain the scientific background and rationale for the investigation being reported **DONE: "See introduction"**
Objectives	3	State specific objectives, including any prespecified hypotheses **See 2nd last paragraph of introduction:** The aim of this study was to determine if levels of blood ILC2s change in the context of a parasitic infection in a human population, thereby providing evidence that ILC2s are important in human parasitic diseases.
**Methods**		
Study design	4	Present key elements of study design early in the paper **Please refer to "Study design and area" and "Study group"**
Setting	5	Describe the setting, locations, and relevant dates, including periods of recruitment, exposure, follow-up, and data collection **See "Study design and area" and "Study group":** This study was conducted in Magaya village in the Murehwa district of the Mashonaland East Province of Zimbabwe (31°91'E; 17°63'S). Samples used within this study were collected between September and November 2008.
Participants	6	*(a)* Give the eligibility criteria, and the sources and methods of case ascertainment and control selection. Give the rationale for the choice of cases and controls **"See study group":** To be included in the study, participants had to meet the following criteria: a) been lifelong residents of the study area, b) not have previously received anti-helminthic treatment, c) be negative for *S. mansoni*, STH, *Plasmodium falciparum* (ensuring that the confounding effects of these parasites were excluded from the study) and HIV, d) have provided at least two urine and two stool samples on consecutive days for parasitological analysis and a blood sample sufficient for PBMC and plasma isolation.
		(b) For matched studies, give matching criteria and the number of controls per case **See "Study group":** 72 people were selected covering an age range of 6-18 years, which were divided into three age groups: 6-9, 10-13, 14-18 years. Within each age group, people were selected to be age and sex between uninfected and infected people, while providing a *S. haematobium* prevalence of 50%. Egg positive samples were chosen to have comparable infection intensities between the three age groups.
Variables	7	Clearly define all outcomes, exposures, predictors, potential confounders, and effect modifiers. Give diagnostic criteria, if applicable Infection with *S. haematobium* was defined by at least one egg positive urine sample. Cell subsets are defined as proportions as, cytokines as concentrations as indicated in the manuscript.
Data sources/ measurement	8*	For each variable of interest, give sources of data and details of methods of assessment (measurement). Describe comparability of assessment methods if there is more than one group: **See "Parasitology section" in Methods:** At least two urine and two stool samples were collected over three consecutive days (between 9am and 1pm). Infection with *S. haematobium* was determined by filtration of 10 mL urine and

		microscopic analysis following the standard urine filtration procedure. Infection with *S. mansoni* and STH were detected using the Kato-Katz method, with the results confirmed in a random subset of stool samples by the formol-ether concentration technique.<br>Up to 20 mL of venous blood was collected into heparinised blood collection tubes and a further five mL into EDTA coated tubes. Heparinised blood was used for the isolation of PBMCs through density gradient centrifugation Lymphoprep™ (Axis-Shield). PBMC were cryopreserved in 10% DMSO (Sigma) and 90% fetal calf serum (Lonza).PBMC were analysed using flow cytometry and Plasma cytokines using ELISA.
Bias	9	Describe any efforts to address potential sources of bias<br>The number of participants post treatment was smaller than pre-treatment numbers. However only paired samples were analysed. The limitations of this analysis are discussed.
Study size	10	Explain how the study size was arrived at<br>**"See study group":** To be included in the study, participants had to meet the following criteria: a) been lifelong residents of the study area, b) not have previously received anti-helminthic treatment, c) be negative for *S. mansoni*, STH, *Plasmodium falciparum* (ensuring that the confounding effects of these parasites were excluded from the study) and HIV, d) have provided at least two urine and two stool samples on consecutive days for parasitological analysis and a blood sample sufficient for PBMC and plasma isolation. 72 people were selected covering an age range of 6-18 years, which were divided into three age groups: 6-9, 10-13, 14-18 years. Within each age group, people were selected to be age and sex between uninfected and infected people, while providing a *S. haematobium* prevalence of 50%. Egg positive samples were chosen to have comparable infection intensities between the three age groups.
Quantitative variables	11	Explain how quantitative variables were handled in the analyses. If applicable, describe which groupings were chosen and why<br>To determine if these differences were consistent across age groups undergoing different schistosome infection dynamics, the population was divided into three age groups, 6-9, 10-13 and 14-18 year olds reflecting rising, peaking and declining infection levels. This is further explained in the discussion.
Statistical methods	12	(*a*) Describe all statistical methods, including those used to control for confounding:<br>See Statistical analysis section
		(*b*) Describe any methods used to examine subgroups and interactions<br>See Statistical analysis section
		(*c*) Explain how missing data were addressed not applicable
		(*d*) If applicable, explain how matching of cases and controls was addressed<br>See Statistical analysis section
		(*e*) Describe any sensitivity analyses not applicable
**Results**		
Participants	13*	(a) Report numbers of individuals at each stage of study—eg numbers potentially eligible, examined for eligibility, confirmed eligible, included in the study, completing follow-up, and analysed<br>Originally a total of 676 participants were recruited into a larger immune-epidemilogical study in Magaya village. Inclusion criteria are provided in the Study group section. After applying point a-d of the inclusion criteria 389 were eligible. Following age restriction (max of 18 years) and availability of PBMCs 143 remained of which 72 were analysed.
		(b) Give reasons for non-participation at each stage<br>The number of participants 6 weeks after treatment was substantially lower than before treatment. Parents or Guardians have withdrawn their children after receiving treatment, which they were allowed without providing reasons (see ethical statement). The youngest age group was in particular affected by this loss.
		(c) Consider use of a flow diagram not applicable
Descriptive data	14*	(a) Give characteristics of study participants (eg demographic, clinical, social) and

		information on exposures and potential confounders provided in Table 1
		(b) Indicate number of participants with missing data for each variable of interest There is one data point missing in IL-4, IL-5, IL-13 (but not TSLP, IL-33) Plasma cytokines due to limited amount of Plasma
Outcome data	15*	Report numbers in each exposure category, or summary measures of exposure
Main results	16	(*a*) Give unadjusted estimates and, if applicable, confounder-adjusted estimates and their precision (eg, 95% confidence interval). Make clear which confounders were adjusted for and why they were included<br>- There was no adjustment of confounders.
		(*b*) Report category boundaries when continuous variables were categorized Age was categorized: To determine if these differences were consistent across age groups undergoing different schistosome infection dynamics, the population was divided into three age groups, 6-9, 10-13 and 14-18 year olds reflecting rising, peaking and declining infection levels. This is further explained in the discussion.
		(*c*) If relevant, consider translating estimates of relative risk into absolute risk for a meaningful time period not applicable

Other analyses	17	Report other analyses done—eg analyses of subgroups and interactions, and sensitivity analyses not applicable
**Discussion**		
Key results	18	Summarise key results with reference to study objectives<br>See Discusssion
Limitations	19	Discuss limitations of the study, taking into account sources of potential bias or imprecision. Discuss both direction and magnitude of any potential bias<br>See Discusssion
Interpretation	20	Give a cautious overall interpretation of results considering objectives, limitations, multiplicity of analyses, results from similar studies, and other relevant evidence<br>See Discusssion
Generalisability	21	Discuss the generalisability (external validity) of the study results<br>See Discusssion
**Other information**		
Funding	22	Give the source of funding and the role of the funders for the present study and, if applicable, for the original study on which the present article is based<br>Wellcome Trust UK (WT082028MA), Thrasher Research Fund. The funders had no role in study design, data collection and analysis, decision to publish, or preparation of the manuscript.

*Give information separately for cases and controls.

Note: An Explanation and Elaboration article discusses each checklist item and gives methodological background and published examples of transparent reporting. The STROBE checklist is best used in conjunction with this article (freely available on the Web sites of PLoS Medicine at http://www.plosmedicine.org/, Annals of Internal Medicine at http://www.annals.org/, and Epidemiology at http://www.epidem.com/). Information on the STROBE Initiative is available at http://www.strobe-statement.org.
