# Supplementary figures and images for: Group 2 Innate Lymphoid Cell Proportions Are Diminished in Young Helminth Infected Children and Restored by Curative Anti-helminthic Treatment
Source: PLoS Negl Trop Dis. 2015 Mar 23;9(3):e0003627. doi: 10.1371/journal.pntd.0003627 (PMC4370749; doi:10.1371/journal.pntd.0003627)

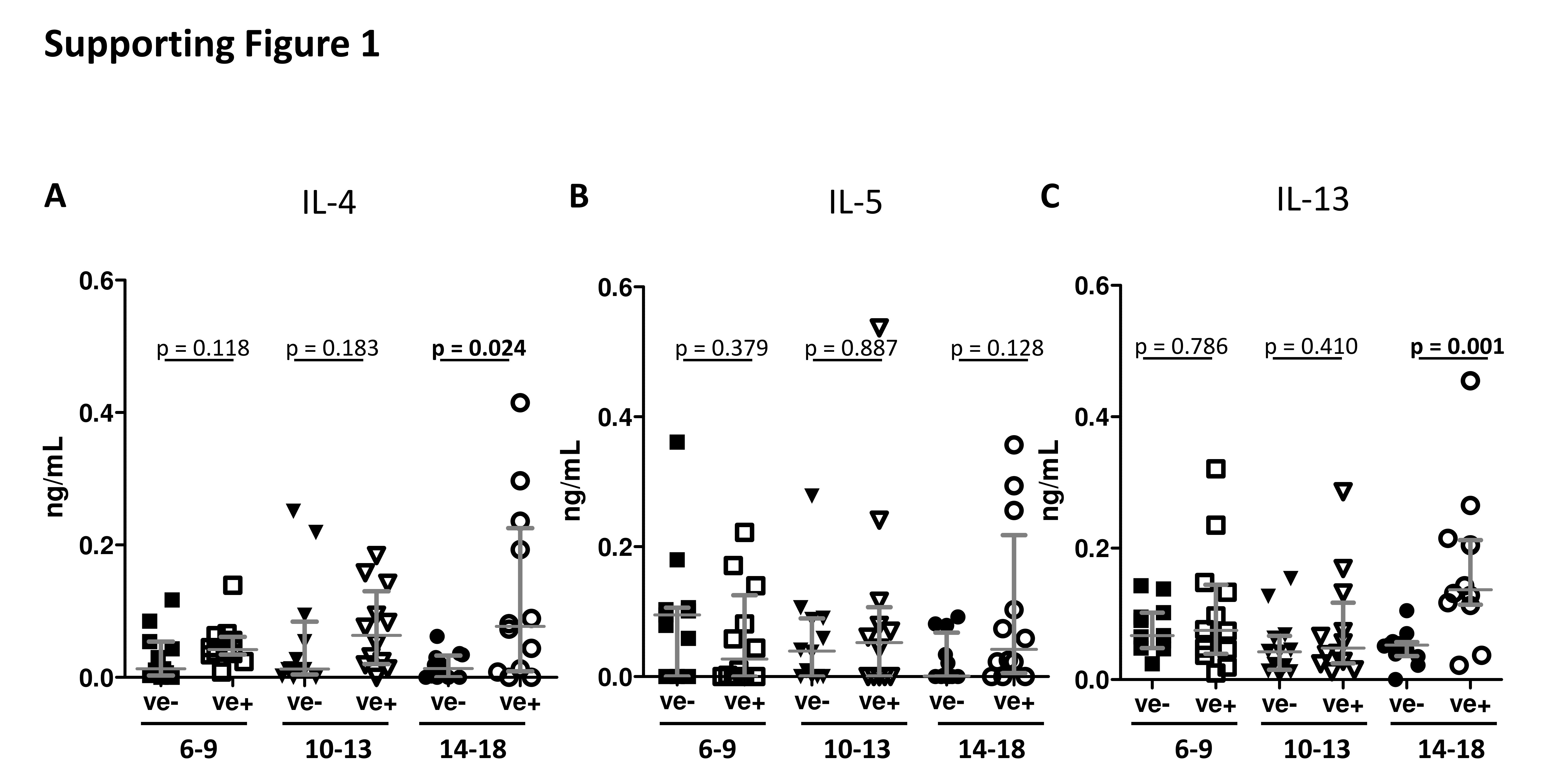

Supplement: S1 Fig — IL-4 (A), IL-5 (B) and IL-13 (C) were analysed by ELISA and data devided by age group and S. haematobium egg negative (ve-, closed symbols) are compared to egg positive (ve+, open symbols). Grey lines indicate Median and the interquartile range and levels were compared using a Kruskal-Wallis test followed by a multiple comparsion. (TIF) [file pntd.0003627.s002.tif]
